# Axially Ligated Mesohemins as Bio-Mimicking Catalysts for Atom Transfer Radical Polymerization

**DOI:** 10.3390/molecules24213969

**Published:** 2019-11-02

**Authors:** Liye Fu, Antonina Simakova, Sangwoo Park, Yi Wang, Marco Fantin, Krzysztof Matyjaszewski

**Affiliations:** Department of Chemistry, Carnegie Mellon University, Pittsburgh, PA 15213, USA; liyef@andrew.cmu.edu (L.F.); simakova@cmu.edu (A.S.); sangwoo82@gmail.com (S.P.); ywang4@andrew.cmu.edu (Y.W.); mfantin@andrew.cmu.edu (M.F.)

**Keywords:** iron porphyrin, heme, ATRPase, iron-mediated ATRP, bio-mimicking catalyst

## Abstract

Copper is the most common metal catalyst used in atom transfer radical polymerization (ATRP), but iron is an excellent alternative due to its natural abundance and low toxicity compared to copper. In this work, two new iron-porphyrin-based catalysts inspired by naturally occurring proteins, such as horseradish peroxidase, hemoglobin, and cytochrome P450, were synthesized and tested for ATRP. Natural protein structures were mimicked by attaching imidazole or thioether groups to the porphyrin, leading to increased rates of polymerization, as well as providing polymers with low dispersity, even in the presence of ppm amounts of catalysts.

Atom transfer radical polymerization (ATRP) is one of the most widely used techniques in the field of reversible deactivation radical polymerization (RDRP) procedures, and it can provide well-defined polymers with predetermined molecular weight, low dispersity, and precisely controlled architecture [1,2,3,4]. ATRP catalysts are predominantly copper-based complexes, due to their extraordinary performance for the synthesis of a broad range of well-defined polymers [5,6]. Nevertheless, developing catalysts with transition metals other than copper is still of great interest [7]. Iron-mediated ATRP has also been extensively studied due to the biocompatibility and low toxicity of iron, which is especially important for biologically relevant systems [8,9,10,11,12]. Although iron-based catalysts offer these potential benefits, their use in ATRP has been limited due to their lower activity and selectivity. Therefore, the design and development of novel iron-based catalysts, which are comparable in activity to conventional copper-based catalysts, and capable of polymerizing a wider range of monomers, are critical to further advancements in this field. 

ATRP is typically performed in organic solvents, but performing ATRP in aqueous media provides several advantages. Water is an environmentally benign solvent, enabling direct polymerization of water-soluble monomers, faster reactions, and polymerization in the presence of biomolecules [13,14,15,16,17]. Several methods for well-controlled Cu-based ATRP in water have been developed, but in the majority of reports a limited number of catalytic systems and a narrow range of monomers have been used [18,19,20]. Control of an ATRP in aqueous media is difficult due to some side reactions including catalyst and chain-end instabilities, as well as the creation of a large equilibrium constant that significantly increases the rate of the polymerization reaction [21,22,23,24].

We have previously reported the synthesis of protein–polymer hybrids using ATRP under biologically relevant conditions, which were designed to sustain the structure of a protein during polymerization while continuing to provide good control of the grafted polymer [25]. In these systems, proteins, appropriately modified with bromoesters or bromoamides, served as macroinitiators for the “grafting from” reaction [26]. Recent publications by Bruns [27,28] and diLena [29,30] have shown that certain proteins/enzymes, such as horseradish peroxidase (HRP), catalase or hemoglobin (Hb), could also serve directly as catalysts for ATRP. These protein-based ATRP catalysts, or “ATRPases”, are proteins comprising heme centers that are able to produce high molecular weight (MW) polymers with dispersity around 1.5~1.6. The relatively high dispersity indicated limited control, plausibly due to insufficient deactivation provided by the bulky protein structures. Nevertheless, these catalytic systems can potentially expand the range of polymerizable monomers because of their different catalyst structure and tolerance to pH variation. However, a major drawback of using proteins as catalysts for ATRP is their sensitivity to reaction conditions and high molecular weight [31]. Therefore, it is necessary to pursue the development of synthetic analogues of natural ATRP enzymes that have enhanced properties, such as the ability to accommodate more stringent reaction conditions with increased mass-to-efficiency ratios of the catalyst complexes, that would allow for a wider range of applications for these biocatalytic systems.

Previously, a successful ATRP of neutral monomers with mesohemin-based catalysts was reported [32]. The hemin was modified with methoxy poly(ethylene glycol) (MPEG) chains to enhance water solubility, and additionally, the vinyl groups were hydrogenated to prevent catalyst copolymerization and consequent incorporation into the polymer chains. Oligo(ethylene oxide) methyl ether methacrylate (OEOMA, *M_n_* = 475) was polymerized under benign aqueous conditions, generating polymers with well-defined molecular weight and low dispersity (<1.2) via activators regenerated by electron transfer (ARGET) ATRP. Acidic monomers, such as methacrylic acid, were directly polymerized with the same catalyst preparing polymers with predetermined MW and acceptable dispersity ~1.5 [33]. Thus, the design and discovery of a novel bio-catalytic systems is still of interest. 

In this paper, two additional mesohemin catalysts were prepared with different ligands, each of which was selected to imitate the axial ligation from amino acid residues present in proteins. The iron center in heme, present in proteins, is often additionally ligated by residues of amino acids such as histidine, cysteine, methionine, or tyrosine [34,35]. Therefore, we chose two types of modification: one with an imidazole moiety to mimic complexation by histidine (Mesohemin-MPEG_550_-Imidazole or MH-MPEG-N), and the other with a thioether moiety to mimic complexation by methionine (Mesohemin-MPEG_550_-Thioether or MH-MPEG-S). The imidazole group has very high complexation affinity towards iron, and thus forms well-defined iron porphyrin complexes. The iron porphyrin complex with thiol has been extensively studied, but we chose to incorporate a thioether to prevent the strong radical transfer property of thiols (Figure 1a).

A series of axially ligated mesohemin complexes were synthesized (Scheme S1), to expand the scope of heme-based catalysts. In this series, one carboxyl group was modified with a poly(ethylene glycol) (PEG) tail and the second carboxyl group was modified with either an imidazole or a thioether via an amidation reaction. Hemin was selected as the starting material for the synthesis of modified iron porphyrins, because hemin is less photosensitive than protoporphyrin IX (hemin without iron), and this strategy did not require an additional step of metal insertion [36]. However, protoporphyrin IX could also have been used for synthesis of modified heme complexes, as it typically provides easier purification and analysis.

The modified mesohemin complexes were characterized by mass spectroscopy, Ultraviolet–visible (UV-Vis) spectroscopy, Infrared (IR) spectroscopy (Figure S1–S8) and cyclic voltammetry (CV; Figure 2a). According to CV measurements, the iron porphyrins formed complexes with varied redox potential E_1/2._, indicating different reactivity. Two new complexes were characterized by less negative E_1/2_values when compared to the fully PEGylated mesohemin, but formed only a single catalytic species, even in the absence of excess bromide [20,37]. Imidazole-modified mesohemin was not significantly affected by the addition of excess bromide ions, but the CV of the thioether-modified mesohemin showed a shift towards a more negative potential (Figure 2b).

To evaluate the feasibility of the new mesohemin catalysts, AGET ATRP’s of OEOMA_500_ were conducted in the presence of MH-MPEG-N/S with the initial polymerization conditions identical to those previously used for MH-(MPEG_550_)_2_ [32]. Polymerization with MH-MPEG-N was more than two times faster than that with MH-(MPEG_550_)_2_, with monomer conversion reaching 76% in only 2h. The final polymer possessed a relatively low dispersity of 1.27, which is slightly higher than that obtained with MH-(MPEG)_2_. One plausible explanation for the increased activity is that it is mainly due to the lower E_1/2_ based on electron donation from the attached imidazole. Polymerization in the presence of thioether-ligated mesohemin (MH-MPEG-S) did not proceed to high conversion, but the final polymer displayed the results of good control, with *M_w_/M_n_* < 1.3 (Figure 3). These reactions suggested that the modifications of mesohemin with axial ligands did provide complexes that could catalyze an ATRP, but additional optimization of reaction conditions needed to be addressed. 

In one such optimization, the addition of less reducing agent in a polymerization catalyzed by MH-MPEG-N resulted in a linear first-order kinetics and linear increase of MW with conversion, with values of MW close to theoretical values. This polymerization resulted in the formation of polymers with dispersity values lower than previously obtained with MH-(MPEG)_2,,_ indicating that conditions had been selected that provided a better controlled polymerization. Additionally, the reaction was faster despite decreased amounts of reducing agent.

In order to verify that the covalent attachment of the imidazole moiety was necessary for the formation of a 1/1 iron porphyrin/imidazole complex, an ATRP with fully PEGylated mesohemin was performed in the presence of free imidazole with a ratio of 1:1 to the iron porphyrin (Table 1, entry 6). This reaction resulted in a slow and poorly controlled polymerization. The final MW of the polymer formed under these conditions was double the theoretically predicted value, indicating inefficient initiation, and M_w_/M_n_ was as high as 1.91. This poorly controlled polymerization could be explained by the fact that two imidazole molecules can complex to the iron porphyrin creating a situation in which a fraction of the catalyst is a hexa-coordinated mesohemin, and another fraction of the catalyst has no imidazole ligands. Since deactivation of a propagating radical cannot occur without the presence of Fe-Br species, the hexacoordinated species consequently results in the loss of deactivation efficiency, thereby providing a poorly controlled polymerization. Therefore, covalent attachment of an imidazole moiety forces preferential formation of a clean 1/1 complex of iron porphyrin and imidazole retaining the Fe-Br bond, which is necessary for performing a well-controlled ATRP.

Because iron porphyrins are highly colored compounds, reaction with a lower concentration of the catalyst would be beneficial for simplification of any desired purification procedures. In the next set of experiments, polymerizations were performed with a 10-fold lower concentration of catalyst. The MH-(MPEG)_2_ did not provide polymers with well-defined *M_n_* when concentration was reduced by a factor of 10 (Table 1, entry 7). However, the axially ligated mesohemins efficiently catalyzed ATRP when their concentrations were decreased by a factor of 10 (Table 1, entries 8, 9). The reaction catalyzed by imidazole-modified mesohemin resulted in formation of a polymer with higher MW than theoretically predicted and relatively high dispersity, reaching a value of 1.5. Nevertheless, the uniform shift in GPC traces toward higher MW indicated that some level of control over the polymerization was attained. Polymerizations conducted in the presence of thioether ligated mesohemin reached higher monomer conversions, over 60%, which was significantly higher than when the catalyst complex was used at higher concentrations. MWs were in good agreement with theoretically predicted values, and the final dispersity of the polymer was less than 1.2 (Figure 4). These results demonstrate that it is possible to use modified hemin complexes as ATRP catalysts at lower concentrations, but further optimization of the amount, and mode of addition, of the reducing agent is required.

In conclusion, a series of bioinspired iron porphyrin-based complexes were designed and successfully utilized as ATRP catalysts. Mesohemin-(MPEG_550_)_2_, prepared from naturally occurring hemin, performed significantly better than hemin itself, or the previously reported hematin-based complex. This can be attributed to the increased solubility of the catalysts in the reaction medium due to the presence of PEG tails. The hydrogenated vinyl bonds prevented copolymerization of the hemin and allowed for faster deactivation in the presence of excess bromide salt. Since this new environmentally benign class of ATRP catalysts showed promise, further modification of mesohemin-based catalysts with different axial ligands were studied. It was found that mesohemin-MPEG_550_, additionally modified with imidazole and thioether units, efficiently catalyzed polymerizations in water at low catalyst concentrations. These bio-mimicking catalyst complexes will be further investigated in polymerization of acidic monomers and for other methods of low ppm ATRP. 

## Figures and Tables

**Figure 1:**
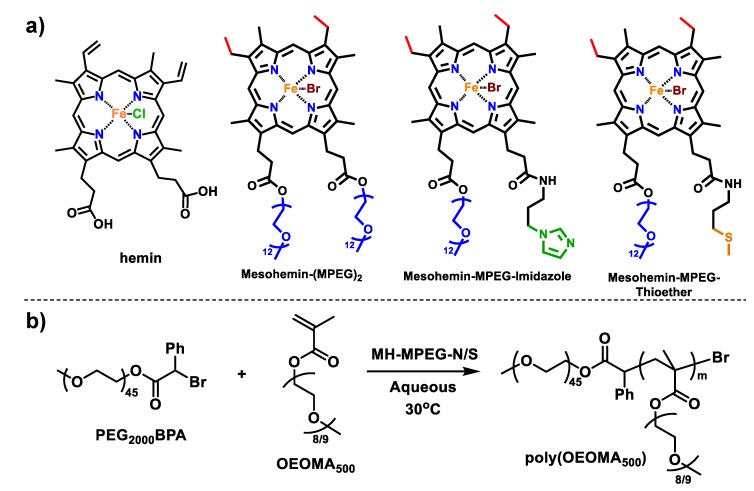
(**a**) Iron porphyrin derivatives used for catalysis of atom transfer radical polymerization (ATRP); (**b**) Scheme of Activator Generated by Electron Transfer (AGET) ATRP of oligo(ethylene oxide) methyl ether methacrylate (OEOMA)_500_.

**Figure 2:**
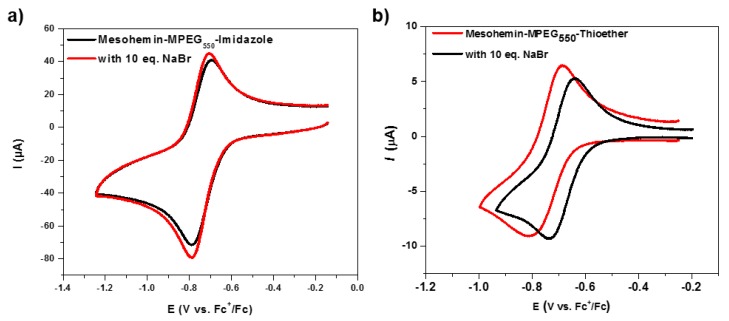
Cyclic voltammogram of (**a**) Mesohemin-methoxy poly(ethylene glycol) (MPEG)_550_-N and (**b**) Mesohemin-MPEG_550_-S, scan rate = 100 mV/s, supporting electrolyte = tetrabutylammonium hexafluorophosphate (TBAPF_6_, 0.1 M in DMF).

**Figure 3:**
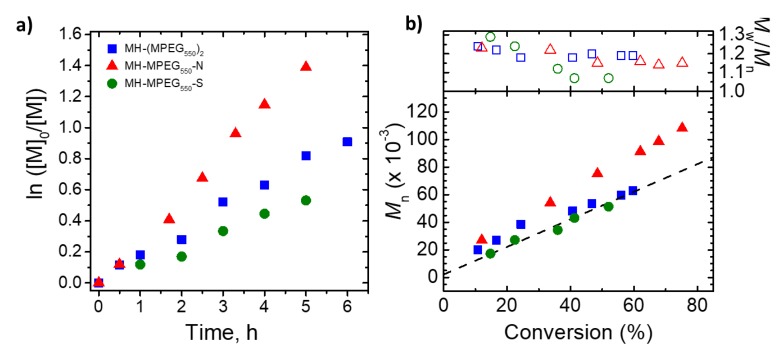
First-order kinetic plots (**a**), evolution of *M_n_* and *M_w_/M_n_* with conversion (**b**) for entry 1-3 in Table 1.

**Figure 4:**
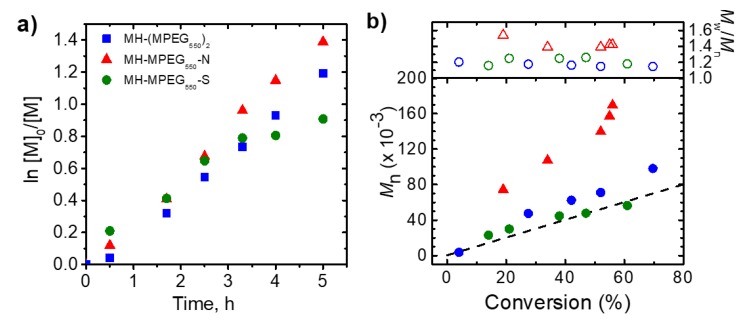
First-order kinetic plots (**a**), evolution of MW and dispersity with conversion (**b**) for entry 7-9 in Table 1.

**Table 1:** Experimental conditions and results of ATRP catalyzed by axially ligated mesohemins ^[a].^

Entry	M/I/RA/Cat	Catalyst	Conv./%, (Time, h)	*M_n,th_* × 10^−3 [b]^	*M_n,GPC_* × 10^−3 [c]^	*M_w_*/*M_n_*
**1**	216/1/1/1	MH-(MPEG)_2_	60 (6)	61	62	1.19
**2**	216/1/1/1	MH-MPEG-N	76 (2.5)	84	76	1.27
**3**	216/1/1/1	MH-MPEG-S	25 (3.5)	27	40	1.28
**4**	227:1:0.3 × 2:1	MH-MPEG-N	75 (5)	83	108	1.16
**5**	227:1:0.3 × 2:1	MH-MPEG-S	41 (5)	43	43	1.07
**6**	216/1/1/1	MH-MPEG_2_ + imidazole	33 (2)	37	78	1.91
**7**	216:1:0.3 × 2:0.1	MH-(MPEG)_2_	70 (5)	72	98	1.17
**8**	216:1:0.3 × 2:0.1	MH-MPEG-N	60 (5)	69	190	1.42
**9**	216/1/0.3 × 2:0.1	MH-MPEG-S	61 (8.7)	61	57	1.18

^[a]^*T* = 30 °C; solvent: H_2_O/DMF = 9/1; [NaBr] = 100 mM; RA = ascorbic acid; I = PEG_2000_BPA; [I] = 2 mM; M = OEOMA_500_; [M] = 20% (*v*/*v*); ^[b]^ *M_n th_* = ([M]_0_/[I]_0_)×conversion×M_monomer_; ^[c]^ *M_n,GPC_* measured by Gel permeation chromatography (GPC) using universal PMMA standards with tetrahydrofuran (THF) as eluent.

## Supplementary Materials

The following are available online at https://www.mdpi.com/1420-3049/24/21/3969/s1. Characterization of aqueous phase catalysts and polymerization results are supplied in the supporting information.
