# Supplementary material for: Axially Ligated Mesohemins as Bio-Mimicking Catalysts for Atom Transfer Radical Polymerization
Source: Molecules. 2019 Nov 2;24(21):3969. doi: 10.3390/molecules24213969 (PMC6864814; doi:10.3390/molecules24213969)
Supplement: Supplementary file 1 [file molecules-24-03969-s001.pdf]

Supporting Information of

Axially Ligated Mesohemins as Bio-mimicking Catalysts for Atom Transfer Radical Polymerization

Liye Fu,[‡] Antonina Simakova,[‡] Sangwoo Park, Yi Wang, Marco Fantin, Krzysztof Matyjaszewski*

Department of Chemistry, Carnegie Mellon University, Pittsburgh, PA 15213

[‡]L.F. and A.S. contributed equally

Materials: All chemicals were purchased from commercial sources, *e.g.*, Sigma Aldrich, TCI, etc. and used as received if not stated otherwise. Hemin (90%, Frontier), Pd/C (10 wt. % loading, matrix activated carbon support, Sigma Aldrich), poly(ethylene glycol) methyl ether ($MPEG_{550}$, $M_{n,avg}$=550), N-(3-Dimethylaminopropyl)-N′-ethylcarbodiimide hydrochloride (EDC·HCl, >99%, Sigma Aldrich), 4-(Dimethylamino)pyridine (DMAP, ≥99%, Sigma Aldrich), 1-(3-aminopropyl)imidazole (≥97%, Sigma Aldrich), 3-(methylthio)propylamine (≥97%, Sigma Aldrich), oligo(ethylene oxide) methyl ether methacrylate ($OEOMA_{500}$, 99%, $M_{n,avg}$=500, Sigma Aldrich) were passed over a column of basic alumina (Fisher Scientific) prior to use to remove inhibitor. Poly(ethylene glycol) bromophenyl acetate ($PEG_{2000}BPA$) was prepared as previously reported in literature.[1]

Gel Permeation Chromatography (GPC): GPC was used to determine number average molecular weight (M_n) and M_w/M_n values. The GPC was conducted with a Waters 515 HPLC Pump and Waters 2414 Refractive Index Detector using PSS columns (SDV 10^2, 10^3, 10^5 Å) in tetrahydrofuran (THF) as an eluent at a flow rate of 1 mL/min at 35 °C. The apparent molecular weights (M_n) and dispersities (M_w/M_n) were determined using linear poly(methyl methacrylate) (M_n = 800 ~ 1,820,000) standards using WinGPC 7.0 software from PSS. The previously reported Mark-Houwink parameters[2] were used for universal calibration using WinGPC 7.0 software from PSS. Conversion was determined using GPC by following the decrease of monomer peak area relative the increase of polymer peak area as previously reported.

Mass spectroscopy: Mass spectra were recorded on a mass spectrometer with a *Varian* Saturn 2100T *MS* with *3900* GC using an EI source. In each case, characteristic fragments with their relative intensities in percentages are shown. Electrospray mass spectra were measured on a Thermo-Fisher LCQ ESI/APCI Ion Trap containing a quadrupole field ion trap mass

spectrometer with electrospray ionization (ESI).

Electrochemical Analysis: All of the cyclic voltammograms (CV) were recorded at 25 °C with a Gamry Reference 600 potentiostat using a standard three-electrode system consisting of a glassy carbon (GC) working electrode, platinum mesh counter electrode, and Ag/AgI/I^- reference electrode. A solution of 0.1 M $TBAPF_6$ supporting electrolyte in 20 mL of DMF was prepared using previously dried reagents. To prepare 1 mM solutions this mixture were added either to 13 mg hemin or 34 mg of mesohemin-$(MPEG_{550})_2$. CV measurements were carried out under a nitrogen atmosphere at a scan rate of 100 mV/s. Potentials were recorded versus a Ag/AgI/I^- reference electrode and the recorded voltammograms were externally referenced to ferrocene/ferrocenium ($Fc^{0/+}$).

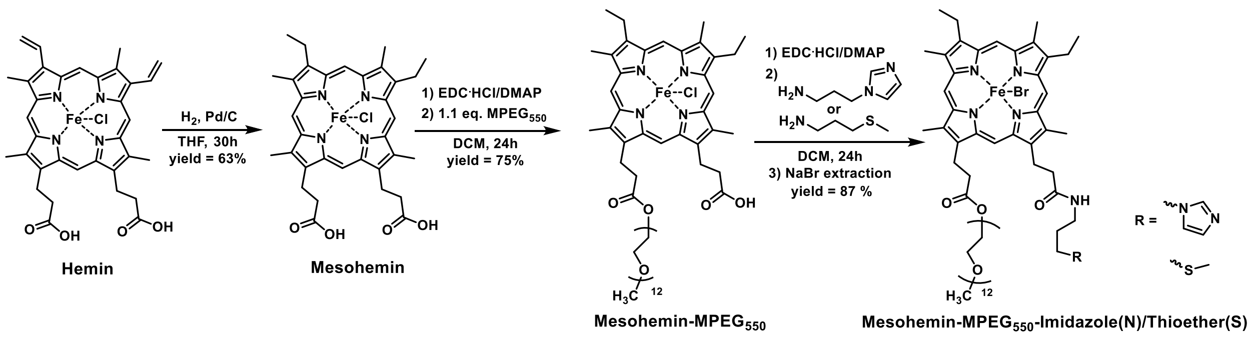

Scheme S1: Preparation of the axially ligated mesohemin derivatives.

Mesohemin synthesis. Mesohemin was synthesized according to the previously reported method for hydrogenation of Hemin (1.1 mmol, 700 mg), Pd/C (15 wt. % to hemin, 105 mg) were mixed in 25 ml Schlenk flask, which was sealed, equipped with balloon and purged with nitrogen. Dry THF (15 ml), obtained from solvent purification system, was added to the dry components through syringe. Balloon was filled with hydrogen, and refilled every 12 h. Reaction was kept for 30 h, then reaction mixture was diluted with 100 ml of methanol, and filtered through layer of celite. Solvent was evaporated under reduced pressure yielding 441 mg (yield = 63%) of mesohemin, which was used for further reaction. Obtained compound was analyzed by ESI-MS. m/z $[M\text{-}Fe+H]^+$ = 567.5, $[M]^+$ = 620.4, $[M+MeOH]^+$ = 651.3, $[2M\text{-}H]^+$ = 1239.4

Mesohemin-$MPEG_{550}$ synthesis. Mesohemin (550 mg, 0.838 mmol) was dissolved in 5 ml of pyridine. Poly(ethylene glycol) methyl ether ($MPEG_{550}$, $MW_{avg.}$=550) (461 mg, 0.838 mmol) and EDC·HCl (177 mg, 0.922 mmol) and DMAP (6 mg, 0.046 mmol) were mixed in 40 mL of DCM in a small flask. Solution with mesohemin was immersed in ice bath, and second mixture was added slowly. The reaction mixture was brought to room temperature and stirred for 24h. After completion of reaction the solution was washed with 0.1 M HCl (2x50 ml), and with

saturated $NaHCO_3$ (2x50 ml). After that mixture was dried with $MgSO_4$ and solvent was removed under reduced pressure. The residue was purified by column chromatography on alumina with chloroform/methanol (9/1) mixture. Fractions were collected, solvent was removed, and the residue was dissolved in 1M HCl in DCM, and washed with saturated $NaHCO_3$. The solution of the product was dried over $MgSO_4$ and solvent was removed under reduced pressure yielding 750 mg of mesohemin-($MPEG_{550}$)$_2$ (75 % yield). The final compound was analyzed by ESI-MS. m/z $[M]^+$: 853.6 – 1470.7 with interval of 44.

Mesohemin-$MPEG_{550}$-N-[3-(1-imidazoyl)propyl]amide (MH-MPEG-N) synthesis. This mesohemin derivative was synthesized in a manner similar to the previously published method. Mesohemin-$MPEG_{550}$ (550 mg, 0.630 mmol), 1-(3-aminopropyl)imidazole (158 mg, 1.260 mmol), and N-(3-dimethylaminopropyl)-N(ethylcarbodiimide hydrochloride (EDC·HCl) (266 mg, 1.390 mmol) and DMAP (8 mg, 0.070 mmol) were mixed in 10 mL of DCM in a small flask while on ice bath. The reaction mixture was brought to room temperature and stirred for 24h. After completion of reaction the solution was washed with 0.1 M HCl (3x10 ml), with saturated $NaHCO_3$ (2x10 ml), and washed with slightly acidic 1M NaBr, and passed through short NaBr column. After that mixture was dried with $MgSO_4$ and solvent was removed under reduced pressure yielding 707 mg of mesohemin-($MPEG_{550}$)$_2$ (87 % yield). The final compound was analyzed by ESI-MS and UV-Vis. λ_{max}: 401, 496, 518, 567 and 621 nm. m/z $[M+Na]^+$: 981.6 – 1605.4 with interval of 44 (M^+).

Mesohemin-$MPEG_{550}$-N-[3-(1-methylthio)propyl]amide (MH-MPEG-S) synthesis.

This derivative was synthesized in a similar manner as imidazole modified version, but reaction mesohemin-$MPEG_{550}$ with 3-(methylthio)propylamine. The final compound was analyzed by ESI-MS and UV-Vis. λ_{max}: 402, 494, and 620 nm. m/z $[M-CH_3+CH_3OH]^+$: 956.6 – 1485.9 with interval of 44.

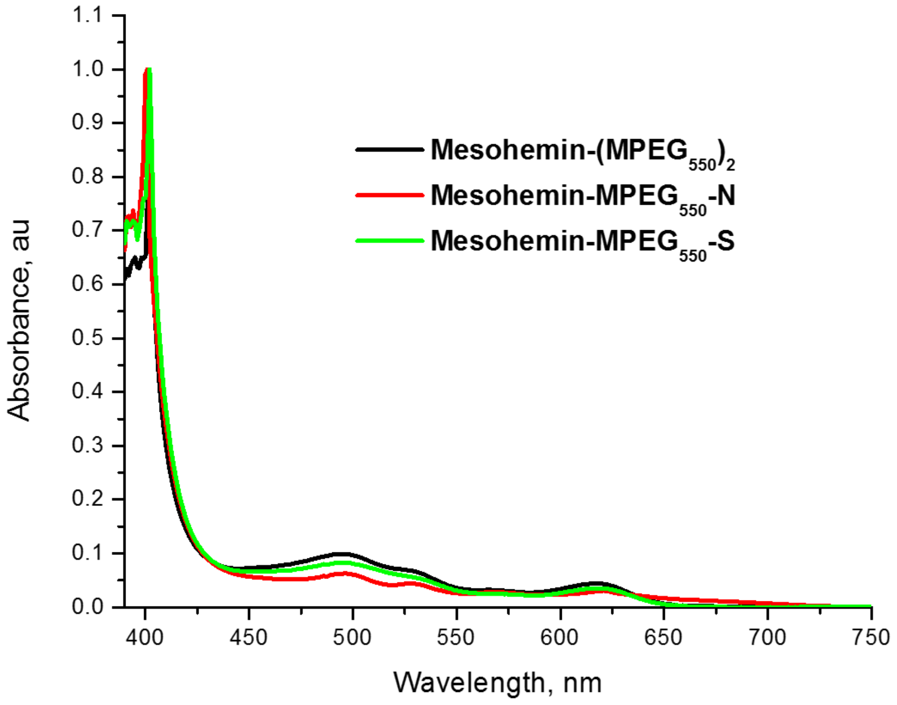

Figure S1: UV-Vis spectra of mesohemin derivatives in methanol (100 µM).

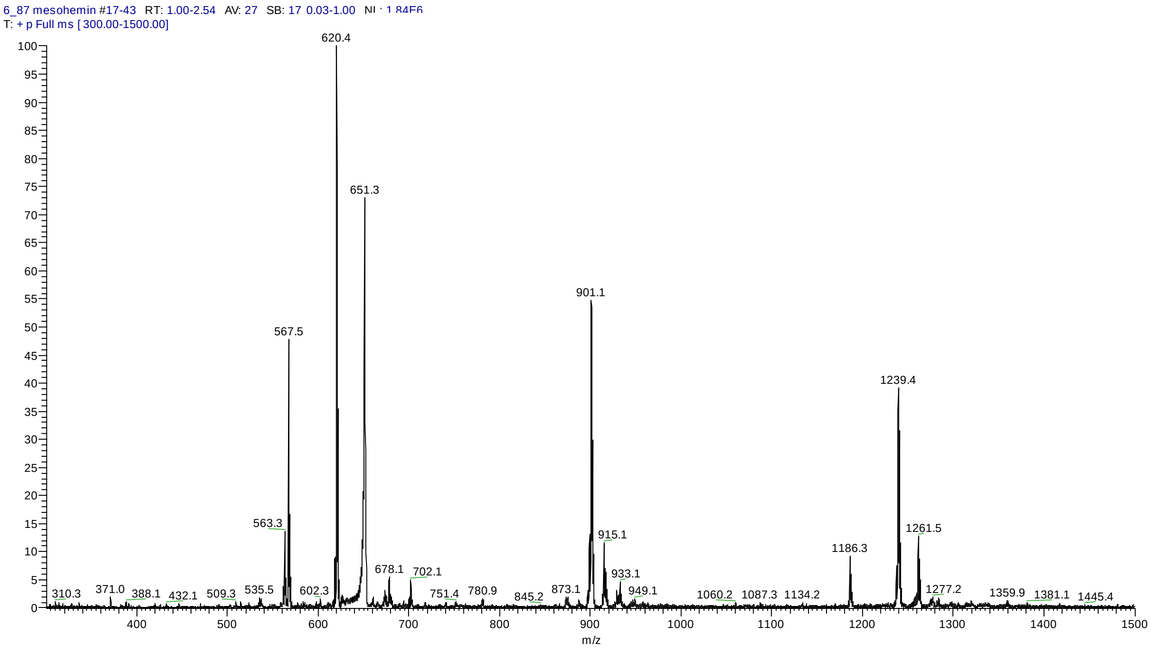

Figure S2: ESI-MS of mesohemin: 250µM in water:methanol = 1:3

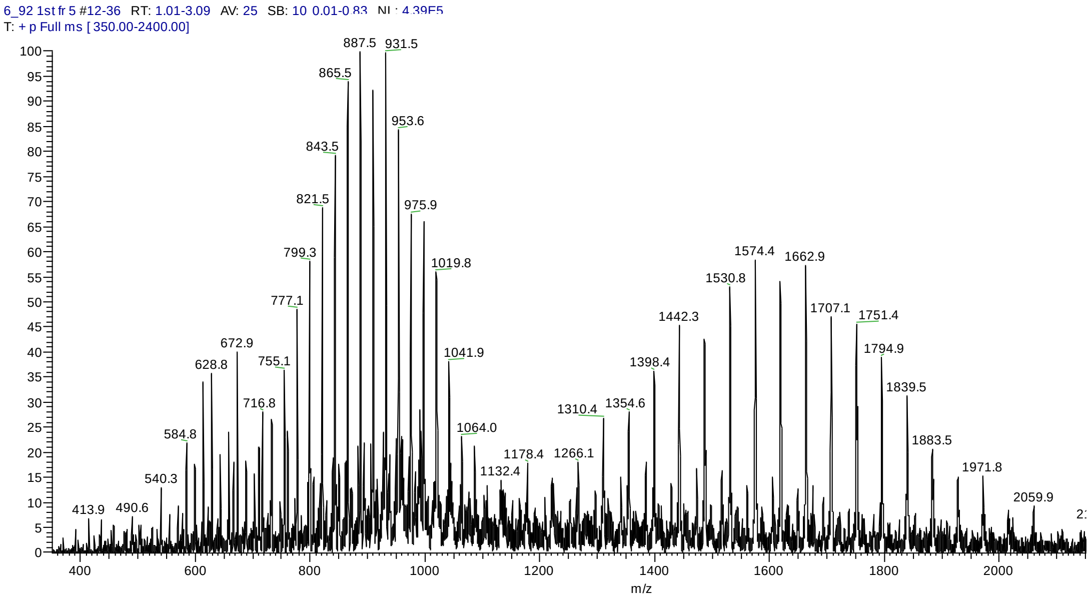

Figure S3: ESI-MS of mesohemin-$(MPEG_{550})_2$: 250µM in water:methanol = 1:3

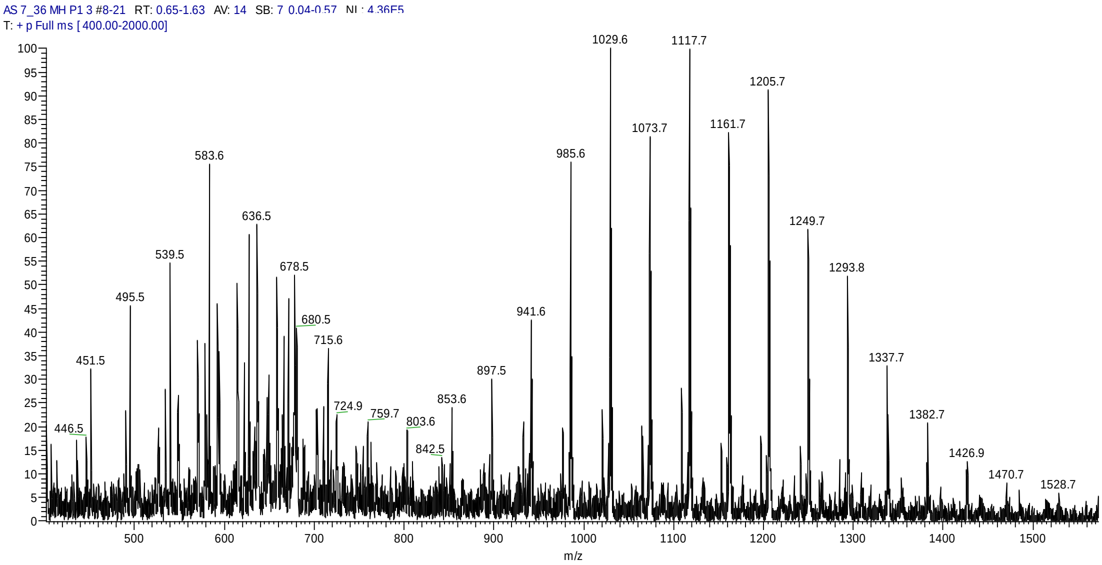

Figure S4: ESI-MS of mesohemin-$MPEG_{550}$: 250µM in water:methanol = 1:3

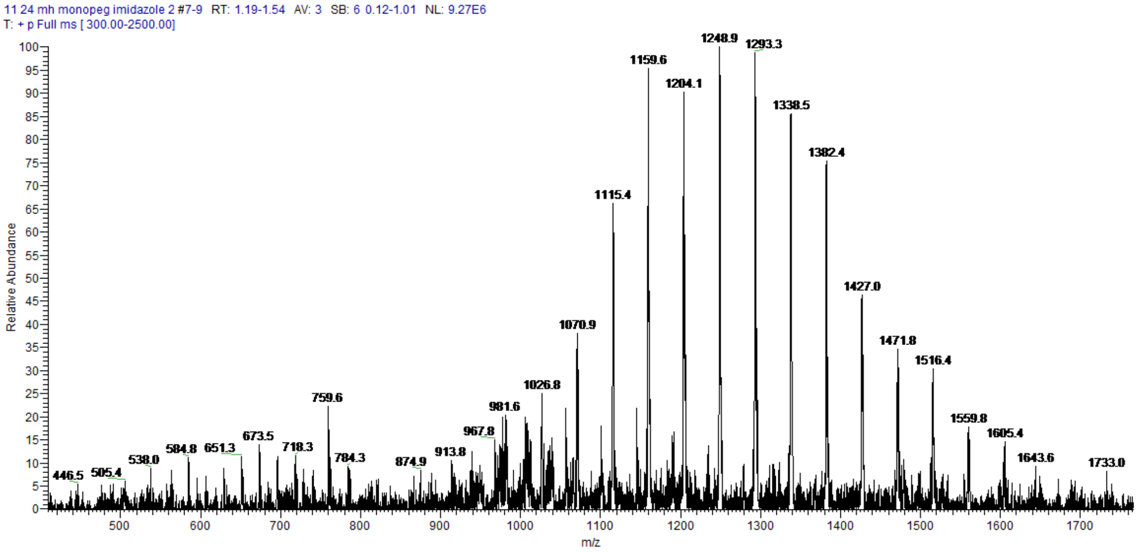

Figure S5: ESI of mesohemin-MPEG$_{550}$-N: 250μM in water:methanol = 1:3

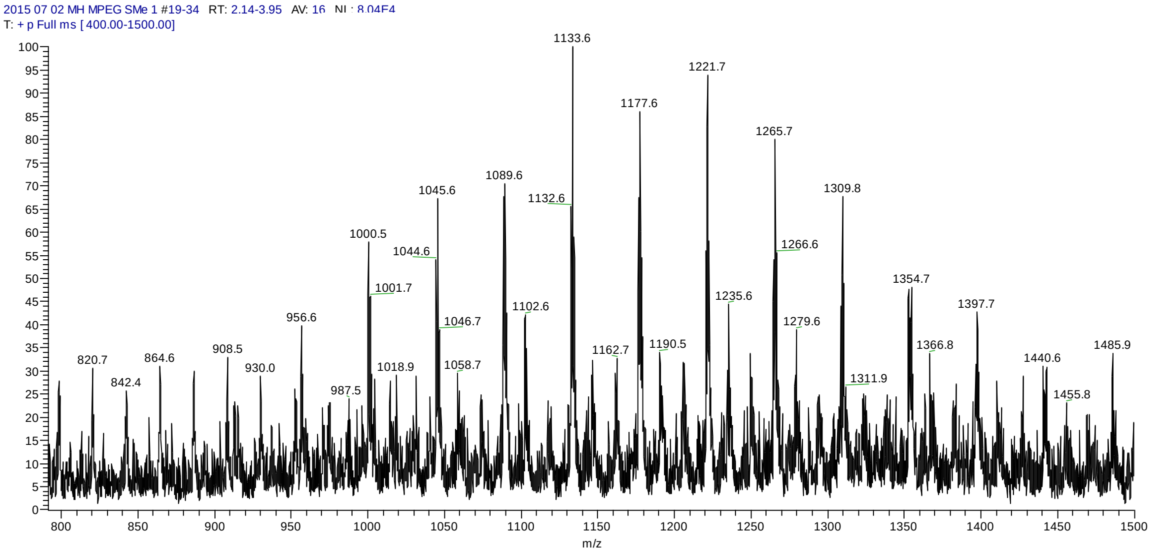

Figure S6: ESI of mesohemin-MPEG$_{550}$-S: 250μM in water:methanol = 1:3

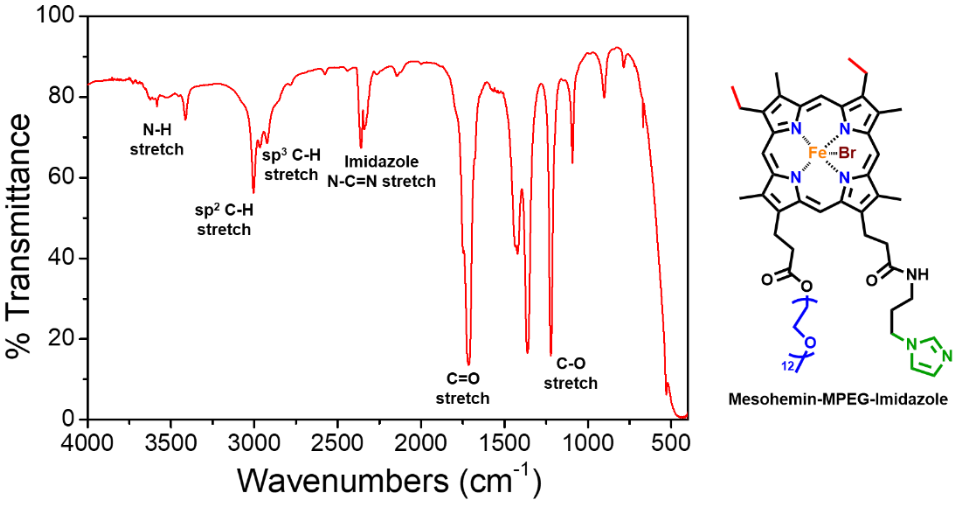

Figure S7: FTIR spectrum of mesohemin-MPEG$_{550}$-Imidazole (MH-MPEG-N).

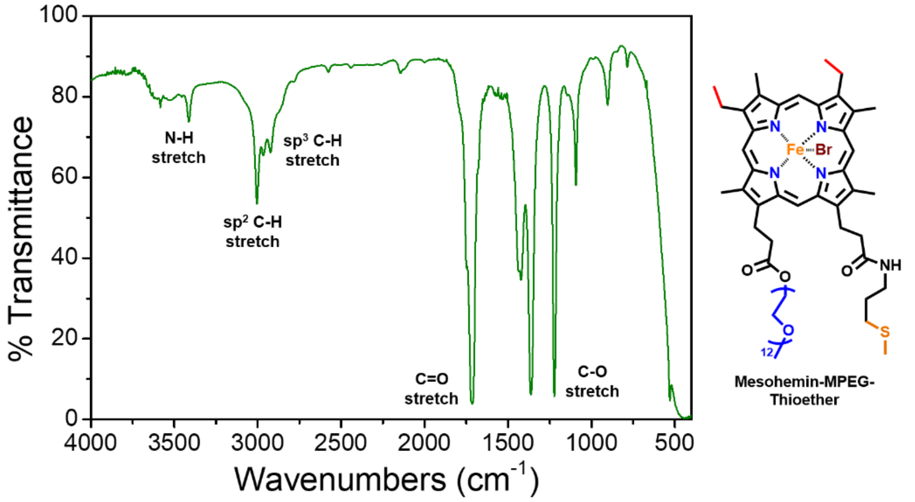

Figure S8: FTIR spectrum of mesohemin-MPEG$_{550}$-Thioether (MH-MPEG-S).

General procedure for synthesis of poly(OEOMA$_{500}$) by A(R)GET ATRP.

A series of aqueous AGET ATRP reactions were carried out and the following procedure describes the conditions selected for a typical polymerization of OEOMA$_{500}$ catalyzed by mesohemin-(MPEG$_{550}$)$_2$. NaBr (60 mg, 0.5 mmol), OEOMA$_{500}$ (1.08 g, 2.27 mmol), mesohemin-(MPEG$_{550}$)$_2$ (17.9 mg, 0.01 mmol) were dissolved in H_2O (3.6 ml) then the mixture was added to a 10 ml Schlenk flask and purged with nitrogen for 1h, then placed in an oil bath at 30 °C. An ascorbic acid solution (100 mM) was purged with nitrogen, and then added to the reaction mixture (0.1 ml). 33 mM stock solution of PEG$_{2000}$BPA in DMF was purged with nitrogen, and then added into reaction mixture (0.3 ml). Samples were taken throughout the reaction for GPC analysis.

Table S1. Redox potentials for various catalysts in DMF

Catalyst	$E_{1/2}$ (V vs. Fc^{+}/Fc) with 10 mM NaBr
**Hemin**	-0.750
**Mesohemin-$(MPEG)_2$**	-0.777
**Mesohemin-MPEG-Imidazole**	-0.735
**Mesohemin-MPEG-Thioether**	-0.725

Scan rate = 100 mV/s, supporting electrolyte = $TBAPF_6$ (0.1 M in DMF), 1 mM of iron porphyrin complex.

REFERENCES

1. Simakova, A.; Mackenzie, M.; Averick, S. E.; Park, S.; Matyjaszewski, K., Bioinspired Iron-Based Catalyst for Atom Transfer Radical Polymerization. *Angewandte Chemie-International Edition* **2013,** *52* (46), 12148-12151.
2. Simakova, A.; Averick, S. E.; Konkolewicz, D.; Matyjaszewski, K., Aqueous ARGET ATRP. *Macromolecules* **2012,** *45* (16), 6371-6379.
